# Predicting Patient Deterioration: A Review of Tools in the Digital Hospital Setting

**DOI:** 10.2196/28209

**Published:** 2021-09-30

**Authors:** Kay D Mann, Norm M Good, Farhad Fatehi, Sankalp Khanna, Victoria Campbell, Roger Conway, Clair Sullivan, Andrew Staib, Christopher Joyce, David Cook

**Affiliations:** 1 The Australian e-Health Research Centre Commonwealth Scientific and Industrial Research Organisation Brisbane Australia; 2 Centre for Health Services Research Faculty of Medicine The University of Queensland Brisbane Australia; 3 School of Psychological Sciences Faculty of Medicine, Nursing and Health Sciences Monash University Melbourne Australia; 4 Sunshine Coast University Hospital Sunshine Coast Hospital and Health Service Birtinya Australia; 5 Clinical Excellence Queensland Queensland Health Queensland Australia; 6 School of Medicine Griffith University Nathan Campas Australia; 7 Metro North Hospital and Health Service Brisbane Australia; 8 Princess Alexandra Hospital Metro South Hospital and Health Service Brisbane Australia; 9 Faculty of Medicine The University of Queensland Brisbane Australia; 10 School of Computer Science Faculty of Science Queensland University of Technology Brisbane Australia

**Keywords:** patient deterioration, early warning scores, digital tools, vital signs, electronic medical record

## Abstract

**Background:**

Early warning tools identify patients at risk of deterioration in hospitals. Electronic medical records in hospitals offer real-time data and the opportunity to automate early warning tools and provide real-time, dynamic risk estimates.

**Objective:**

This review describes published studies on the development, validation, and implementation of tools for predicting patient deterioration in general wards in hospitals.

**Methods:**

An electronic database search of peer reviewed journal papers from 2008-2020 identified studies reporting the use of tools and algorithms for predicting patient deterioration, defined by unplanned transfer to the intensive care unit, cardiac arrest, or death. Studies conducted solely in intensive care units, emergency departments, or single diagnosis patient groups were excluded.

**Results:**

A total of 46 publications were eligible for inclusion. These publications were heterogeneous in design, setting, and outcome measures. Most studies were retrospective studies using cohort data to develop, validate, or statistically evaluate prediction tools. The tools consisted of early warning, screening, or scoring systems based on physiologic data, as well as more complex algorithms developed to better represent real-time data, deal with complexities of longitudinal data, and warn of deterioration risk earlier. Only a few studies detailed the results of the implementation of deterioration warning tools.

**Conclusions:**

Despite relative progress in the development of algorithms to predict patient deterioration, the literature has not shown that the deployment or implementation of such algorithms is reproducibly associated with improvements in patient outcomes. Further work is needed to realize the potential of automated predictions and update dynamic risk estimates as part of an operational early warning system for inpatient deterioration.

## Introduction

### Background

In recent years, proactive clinical processes have been developed to target timely and appropriate care for deteriorating or high-risk patients. Emergency responses such as *Rapid Response Systems* have been implemented with the aim to intervene and avoid preventable death, cardiac arrest, or transfer to an intensive care unit (ICU) in adult [1-3] and pediatric [4,5] patients. These systems have evolved to consist of a recognition (afferent) limb, commonly known as early warning scores (EWSs), and a response (efferent) limb (escalation and intervention). The responders rely on an accurate recognition limb, which in turn relies on a combination of empirical rules, statistical models, and clinical judgment to recognize deterioration. Initial EWSs were limited to vital signs, as these were the only routinely collected physiological data available for analysis in real time. The EWSs are currently available as paper or digital tools and vary significantly in their modeling, design, and escalation guidance [3,6-10].

The growth of rich, detailed, and dynamic clinical digital documentation in electronic medical records (EMRs) raises the possibility of using a broader range of clinical data, including pathology and diagnoses. An EMR collects the detailed phenotype of the patient in real time. Data collected, such as previous history, comorbidities, and demographic descriptors, are static during an admission. Observations of dynamic processes such vital signs, clinical measurements, imaging, and laboratory results that document biological and pathological processes are also recorded and continuously updated. Further evolving diagnoses, events, and interventions (eg, operations and drugs that are administered) capture the changing status of a patient. Finally, a rich source of dynamic information lies in the metadata: the timing, frequency, and location of actions and observations that occur to the patient and patient movements in the system. These data enable refined predictive models and more effective, patient-specific treatments. To create clinical decision support, these data must be analyzed and risk interpreted, and then critically, the clinical decision support communicating this real-time risk needs to be engineered back into the routine clinical workflows of the clinicians caring for the patient.

There is a diversity of models for predicting patient deterioration. Some risk estimates are static systems that identify high-risk patients at the time of diagnosis and allow triage of patients to a higher intensity care destination. Other approaches use vital sign observations to maintain an up-to-date risk evaluation. Typically, these dynamic systems either identify an extreme singular derangement or use weighted sums of a few vital signs and their variation from normal values. In both situations, the likelihood of deterioration and poor outcome anticipated with worsening values is increased. In some cases, these models have been developed and validated only for specific and narrow patient groups [11-13], whereas other general models are applicable to wider adult or pediatric ward patients [3-5,10,14].

### Objectives

The aim of this review is to identify studies conducted within a general hospital setting that have attempted to develop prediction algorithms for detecting a deteriorating ward patient in real time, based primarily on routinely collected EMR data. This review includes model statistical validation and, where available, the results of digital hospital implementation of new or existing prediction models or rule-based systems for predicting patient deterioration. A secondary aim is to review those successful examples for common data elements, approaches, and statistical or machine learning techniques that were associated with successful clinical use.

## Methods

This review followed the recommendations of the Center for Reviews and Dissemination [15] and PRISMA (Preferred Reporting in Systematic Reviews and Meta-Analyses) [16].

### Data Sources and Search Strategy

A systematic search of PubMed, Scopus, Web of Science, IEEE Xplore, and ACM Digital Library using a combination of controlled vocabulary (eg, MeSH [Medical Subject Headings] terms) and free text keywords was conducted. The search strategy was first developed for PubMed, guided by the recommendations of Hausner et al [17] and Fatehi et al [18], and transposed to other databases. Across databases, free text keywords remained the same, but controlled vocabulary was mapped where possible (eg, from MeSH to Index Terms). The search was limited by language (English), date of publication (January 1, 2008, to June 30, 2020) and type of publication (original papers).

### Screening and Study Selection

Reports that have developed prediction algorithms for detection of clinical patient deterioration in real time, primarily based on routinely collected patient data, were identified. The included studies had to (1) use any kind of (electronic) patient record, (2) use an early warning tool for patient deterioration, (3) use of a system that was dynamic, or observations of a patient over time, and (4) document the model statistical accuracy and performance. The studies were peer reviewed and published in journals or conference proceedings. The focus of this review was on tools in a general hospital ward and in a real time setting. Excluded studies were those conducted solely in a critical care or an emergency department setting, those limited to a single diagnosis or organ dysfunction patient cohort, those that used a static time point or an observation to assess risk of deterioration, and those that were qualitative with no quantitative assessments.

The results of electronic database searches were exported into an EndNote [19] library, and duplicates were removed. Title and abstract screening was coordinated on Rayyan web application (Qatar Computing Research Institute) [20]. Two independent reviewers screened titles and abstracts for relevance, and unresolved differences were adjudicated by a third reviewer. Full texts of remaining papers were assessed by 2 independent reviewers against the inclusion and exclusion criteria. Unresolved differences were adjudicated by a third reviewer. Where more than one publication was found for the same project, the papers were grouped, and the publication with the most comprehensive findings was included.

### Data Extraction and Synthesis

An electronic web form was developed according to the aims of this review and was used for data extraction from full-text papers. Data extraction was conducted by one reviewer and checked by a second reviewer. Owing to the heterogeneity of the included studies in terms of study designs, aims, and outcome measures, it was not possible to conduct a meta-analysis. Thus, a narrative approach was adopted for data synthesis. An assessment of study quality, including risks of bias, was conducted using PROBAST (Prediction Model Risk of Bias Assessment Tool; UMC Utrecht) [21] and ROBINS-I (Risk of Bias in Non-Randomised Intervention Studies; Cochrane) tool [22].

## Results

### Overview

**Figure 1:**
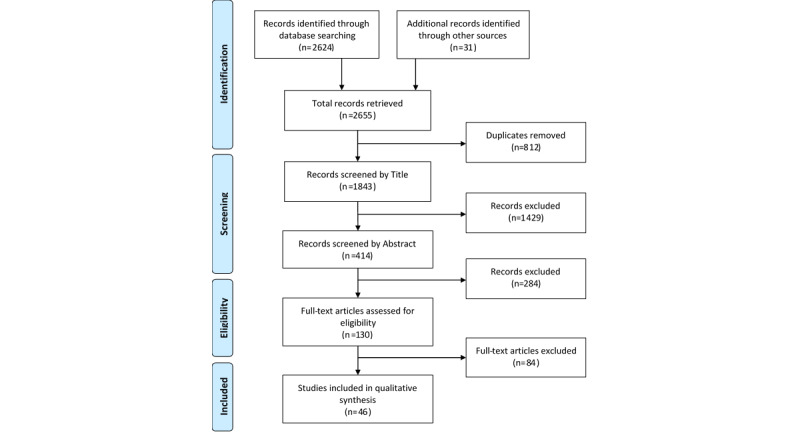
PRISMA (Preferred Reporting Items for Systematic Reviews and Meta-Analyses) flow diagram of study selection.

The initial electronic search of five databases yielded 2624 records, and an additional 31 records were identified from other sources (Figure 1). After removing duplicates, 1843 unique records remained for eligibility assessment. Following the screening of titles and abstracts, 130 papers were deemed relevant, and their full texts were obtained. After a full-text inspection, 46 papers met the eligibility criteria and were included in this review.

### Characteristics of the Studies

Of the 46 papers in this review, the majority (37/46, 80%) were retrospective studies that attempted to develop and/or evaluate the performance of a prediction model or compare the performance of a number of predictive models using historical patient data [23-59]. One study reported both the retrospective development of a prediction model and a prospective observational study of the developed model [60]. The remaining studies included one randomized controlled trial [61], 4 before-after (implementation) studies [62-65], and 3 prospective observational studies [66-68].

### Overview of Studies

Studies from the same setting and institution were grouped and ordered according to the chronological model development, evaluation, and implementation, if available.

Of the identified studies, 6 described a body of research undertaken by researchers from Kaiser Permanente in California, as well as the University of Chicago [34,35,40,44,62,63] (Table 1). Kaiser Permanente is a large integrated managed care organization in the United States and began the deployment of EMRs in its hospitals in 2006. Escobar et al [35] reported the development of the early detection of impending physiological deterioration algorithm, which was further developed and subsequently named the Advanced Alert Monitor [44] and the early detection of impending physiological deterioration version 2 algorithm [40]. These models were developed to predict the risk of unplanned ICU transfer or mortality, using historical data of hundreds of thousands of patients retrieved from the Epic system, have also been retrospectively tested in a simulation study for feasibility [40], piloted in 2 hospitals [62], and more recently implemented as the Advanced Alert Monitor program and evaluated across the remaining Kaiser Permanente Hospitals [63,69,70].

**Table 1:** Studies undertaken on Kaiser Permanente Hospitals.

Author and year	Study design	Settings	Study aim	Model type or used	Outcome measure	Key findings
Escobar et al, 2012 [35]	RS^a^: tool development	102,422 hospitalizations; 14 KP^b^ hospitals; 2006-2009	To develop a model for the prediction of unplanned ICU^c^ transfer using EMR^d^ data	Pooled logistic regression models	Transfer to ICU or death	EMR-based detection of impending deterioration outside the ICU is feasible. The overall performance of the model incorporating physiology, diagnosis, and longitudinal data was superior to MEWS^e^. Model *c* statistic 0.85, validation model 0.78
Escobar et al, 2013 [34]	RS: tool development	391,584 hospitalizations; 248,383 patients; 21 KP hospitals; 2008-2011	To develop a risk adjustment methodology applicable to all hospitalized patients	Pooled logistic regression models	Death	Risk adjustment of hospital mortality using EMR is feasible. Incorporation of physiological data increased model discrimination and explanatory power. Model *c* statistic 0.80, validation model 0.88
Kipnis et al, 2016 [44]	RS: tool development and evaluation	649,418 hospitalizations; 374,838 patients; 21 KP hospitals; 2010-2013	To describe the development and performance of an automated EWS^f^ based on EMR data: The AAM^g^	Discrete time logistic regression	Transfer to ICU or death	The AAM had better performance compared with NEWS^h^ and eCART^i^ in all metrics and prediction intervals (AUC^j^ 0.82). Around half the alerts triggered occurred within 12 hours of the event and almost two-thirds within 24 hours.
Escobar et al, 2016 [62]	Prospective study: tool implementation	2 KP hospitals	To detail technical and operational challenges of deploying an EWS	AAM	Pre- and postimplementation metrics	The pilot implementation in 2 hospitals was successful and further deployment to other hospitals will go ahead.
Hu et al, 2018 [40]	RS: tool simulation and feasibility	174,632 hospitalizations; 21 KP hospitals	To evaluate the impact of proactive transfer to ICU based on EDIP2^k^ score, on mortality rate and LOS^l^	EDIP2	Transfer to ICU or death	Proactively transferring of the most severe patients could reduce mortality rates without sacrificing other patient outcomes.
Escobar et al, 2020 [63]	RS: tool implementation and evaluation	548,838 hospitalizations; 326,816 patients; 21 KP hospitals	To evaluate a staggered deployment of an automated predictive model; identifying patients at high risk for clinical deterioration.	AAM	Pre- and postimplementation outcomes: transfer to ICU, 30-day mortality, LOS, favorable status	30-day mortality after an alert was lower in the intervention compared with control (three deaths avoided per 1000 eligible patients). The intervention was also associated with lower incidence of ICU admission, higher percentage of patients with favorable status 30 days after alert, shorter LOS, and longer survival.

^a^RS: retrospective study.

^b^KP: Kaiser Permanente.

^c^ICU: intensive care unit.

^d^EMR: electronic medical record.

^e^MEWS: Modified Early Warning Score.

^f^EWS: early warning score.

^g^AAM: Advanced Alert Monitor.

^h^NEWS: National Early Warning Score.

^i^eCART: electronic Cardiac Arrest Risk Triage.

^j^AUC: area under the receiver operating characteristic curve.

^k^EDIP2: early detection of impending deterioration version 2.

^l^LOS: length of stay.

Another group of papers from the University of Chicago program was based on experiments using the electronic patient records from a number of hospitals in the Chicago area [29-33,37,66] (Table 2). Churpek et al [29] in several papers reported the development and validation of the electronic Cardiac Arrest Risk Triage score and other tools for predicting cardiac arrest and ICU transfer in Chicago (Table 2). They also showed that mortality and cardiac arrest were easier to predict than ICU transfer [29], and machine learning methods were more accurate than logistic regression for predicting patient deterioration [32].

**Table 2:** Studies conducted in Chicago hospitals.

Author and year	Study design	Setting	Study aim	Model type or used	Prediction event	Key findings
Churpek et al, 2012 [31]	RS^a^: tool development and evaluation	47,427 patients, 1 hospital, 2008-2011	To develop a CART^b^ score and compare with the MEWS^c^	Logistic regression	CA^d^ or transfer to ICU^e^	The CART score more accurately predicted cardiac arrest than the MEWS. Model AUC^f^ 0.84
Churpek et al, 2013 [29]	RS: tool development	59,643 patients, 1 hospital, 2008-2011	To assess the impact of outcome selection on the performance of prediction algorithms	Logistic regression (4 models)	CA, transfer to ICU, death, all combined	Mortality is the easiest outcome to predict (AUC range 0.73-0.82), and ICU transfer is the most difficult.
Churpek et al, 2014 [30]	RS: tool development	59,301 patients, 1 hospital, 2008-2011	To derive and validate a prediction model for CA	Logistic regression	CA and transfer to ICU	The model can simultaneously predict the risk of CA and ICU transfer and was more accurate than ViEWS^g^. Model AUC 0.88 for CA, 0.77 for ICU
Churpek et al, 2014 [33]	RS: tool development	269,999 admissions, 5 hospitals, 2008-2011	To develop and validate eCART^h^ score using commonly collected EMR^i^ data	Survival analysis	CA, transfer to ICU, or death	eCART score was more accurate than MEWS for detecting CA, ICU transfer, or death. Model AUC 0.83 for CA, 0.75 for ICU transfer, 0.93 for death and 0.77 all combined
Somanchi et al, 2015 [56]	RS: tool development	133,000 patients, 4 hospitals, 2006-2011	To develop a prediction model for Code Blue, using EMR data, and compare with MEWS	SVM^j^ and logistic regression	Code blue event in the next x hours	The model was able to predict Code Blue with ~80% recall and 20% false positive rate 4 hours ahead of the event. It out-performed MEWS.
Churpek et al, 2016 [32]	RS: tool development and evaluation	269,999 patients, 5 hospitals, 2008-2013	To compare the accuracy of different techniques for detecting clinical deterioration on the wards	Logistic, decision trees, SVM, K-NN^k^, neural net, MEWS	CA, transfer to ICU, or death	This multicenter study showed that several machine learning methods can more accurately predict clinical deterioration than logistic regression.
Kang et al, 2016 [66]	Prospective study: feasibility study	3889 admissions, 3 wards, 2013-2014	To assess the feasibility of a real-time risk stratification tool	eCART	CA, transfer to ICU, or death	eCART score identified more CA and ICU transfers, many hours in advance, compared with standard RRT^l^ activation.
Green et al, 2018 [37]	RS: tool evaluation	107,868 admissions, 5 hospitals, 2008-2013	To compare the BTF^m^ calling criteria to MEWS, NEWS^n^ and eCART score	BTF, NEWS, MEWS, eCART	CA, transfer to ICU, or death (24 hours)	eCART was more accurate than BTF, MEWS, NEWS for predicting the composite outcome of CA, ICU transfer and death. eCART AUC 0.80 (0.79-0.80)
Bartkowiak et al, 2019 [25]	RS: tool evaluation	32,537 admissions, 1 hospital, 2008-2016	To assess the accuracy of three EWS^o^ postoperatively	NEWS, MEWS, eCART	CA, ICU transfer or ward, or death	The eCART score was the most accurate followed by MEWS. Maximum respiratory rate was the most predictive vital sign.
Mayampurath et al, 2019 [48]	RS: tool development	115,825 admissions, 1 hospital, 2008-2016	To develop a model from visual timelines to predict mortality	Convolutional neural network	Death	The model was more accurate than MEWS and SOFA^p^, validation model AUC 0.91, and visual timelines enabled interpretation of a deep neural network.

^a^RS: retrospective study.

^b^CART: Cardiac Arrest Risk Triage.

^c^MEWS: Modified Early Warning Score.

^d^CA: cardiac arrest.

^e^ICU: intensive care unit.

^f^AUC: area under the receiver operating characteristic curve.

^g^ViEWS: VitalPAC Early Warning Score.

^h^eCART: electronic Cardiac Arrest Risk Triage.

^i^EMR: electronic medical record.

^j^SVM: support vector machine.

^k^K-NN: K-nearest neighbors.

^l^RRT: rapid response team.

^m^BTF: Between the Flags.

^n^NEWS: National Early Warning Score.

^o^EWS: early warning score.

^p^SOFA: Sequential Organ Failure Assessment.

Several other studies have evaluated screening tools such as the National Early Warning Score, Modified Early Warning Score, Rothman Index, and Sequential Organ Failure Assessment, for the early warning and detection of patient deterioration in hospital settings across different countries [26,39,41,50,52,54,57,59,64,65,67] (Table 3). The evaluation included applying tools retrospectively on historical clinical data to assess the feasibility of future use as well as assessing tools prospectively alongside the standard clinical systems. The studies of screening tools reported differing results of both good and poor predictive accuracy and usefulness for escalation of care through medical emergency team (MET) or rapid response team (Table 3).

Of the studies detailing the implementation of scoring tools across institutions [50-52,61,62,64,65], high-risk patients were appropriately identified as aiding in clinical response (Table 4). However, when comparing intervention and control patient cohorts, differing results were seen with either significant reductions or no impact on the assessed deterioration events.

Within the reviewed papers, deterioration prediction methodologies included single and multiparameter scoring tools, such as the National Early Warning Score and Modified Early Warning Score, as well as statistical and machine learning methods. Single and multiparameter scores were derived from a set of vital sign threshold derangements (Tables 3 and 4). The statistical and machine learning methods included logistic regression, survival models, Cox regression, Gaussian process regression, Markov models, decision trees (random forest and gradient boosted trees), K-nearest neighbor, support vector machine, and neural networks (Tables 1 and 2, and Multimedia Appendix 1 [23,24,27,28,36,38,42,43,45-47,49,51,53,55,58, 60,68]). Most of these models attempted to account for changes in physiologic measures over time using novel model frameworks, for example, taking a sliding time window looking forward or backward in time to predict outcomes.

Across all the studies, models of greater complexity and statistical and machine learning methods were shown to have superior performance in predicting deterioration than scoring tools (Multimedia Appendix 1). For example, there was timelier detection and earlier warning of high deterioration risk in more complex models. Furthermore, the discrimination of patient deterioration using statistical and machine learning methods (as assessed with the area under the receiver operating characteristic curve or *c* statistic) outperformed conventional tools.

All patient deterioration prediction tools used vital sign measures, most commonly blood pressure, heart rate, respiratory rate, temperature, oxygen saturation, and a level of consciousness measure (Multimedia Appendix 2, Tables S2 and S3 [23-39,41-45,47-61,63-65,67,68]). In addition, most of the models included basic patient demographic data, such as age and gender, as well as administrative measures, such as admission status, time since admission, length of stay, and patient location. Many of the models attempted to incorporate various pathology or laboratory test results where available, noting that they would experience some level of time delay. Composite indices or scores for severity of illness, longitudinal comorbidities, and combined laboratory results were often included in models where available. The more complex models considered higher-level features of their data such as the frequency, change, minimum, maximum, moving average, and patterns of physiological measures over time [23,24,38,42,45,56,58].

Of the studies that reported missing data, the majority were filled by propagating a previous value carried forward if a current value within a set time window was not measured. If no prior value was available, values were imputed with a representative value such as a population-based estimate or normal value. In models that consider dynamic irregularly sampled physiologic time series data, Gaussian process models were used to deal with sparsity in the data [23,24,55].

Overall, the quality of the studies included in this review was high, with low and unclear risks of bias and concern for the applicability of prediction models in addressing our review questions (Multimedia Appendix 2, Table S2). The majority of prediction model studies appropriately selected model data for inclusion, assessed model predictors and outcomes, approached model development, and adequately tested models. Studies assessed as unclear were because of ambiguous details in reporting the number of participants or samples per derivation and validation data sets, handling of missing data, and lack of detail in model validation. The study quality of the implementation studies we identified was also high, with low to moderate risk of bias identified (Multimedia Appendix 2, Table S4). Moderate concerns were because of the lack of adjustment for potential confounding, participant selection, and lack of detail for handling of missing data.

**Table 3:** Retrospective studies evaluating scoring tools.

Author, year, and country	Settings	Study aim	Scoring tools	Prediction event	Key findings
Lighthall et al, 2009 [67], United States	1089 patients, 1 hospital, 2006	To evaluate vital signs and association with critical events	MET^a^ call criteria	CA^b^, ICU^c^ transfer or death	Even a single recording of an abnormal vital sign increases the risk of critical events in hospitalized patients.
Huh et al 2014, [41], Korea	3030 events, 1 hospital, 2008-2010	To evaluate the efficacy of screening triggered alerts for MET management	Medical alert system criteria	MET activation	The automatic alert system triggers, along with a skilled intervention team, were successful in managing the MET
Romeo-Brufau et al, 2014 [54], United States	34,898 patients, 2 hospitals, 2011	Comparative analysis of the performance of common EWS^d^ methods and how they would function if automated	MEWS^e^, SEWS^f^, GMEWS^g^, Worthing, ViEWS^h^, NEWS^i^	Resuscitation call, RRS^j^ activation or ICU transfer	The evaluated scores did not offer good predictive capabilities for an automated alarm system. Positive predictive values ranged from <0.01-0.21, and sensitivity ranged from 0.07-0.75.
Yu et al, 2014 [59], United States	328 cases, 328 controls, 1 hospital, 2009-2010	To compare the ability of 9 risk prediction scores in detecting clinical deterioration among non-ICU ward patients	SOFA^k^, PIRO^l^, ViEWS, SCS^m^, MEDS^n^, MEWS, SAPS II^o^, REMS^p^, APACHE II^q^	Critical care consult, ICU transfer or death	Prediction scores can be used to estimate a ward patient’s risk of clinical deterioration, with good discriminatory ability comparable with that of existing track-and-trigger systems. 0-12 hours before clinical deterioration, 7 of 9 scores performed with acceptable discrimination (AUC^r^>0.70).
Wengerter et al, 2018 [57], United States	217 cases, 868 controls, 1 hospital, 2013-2015	To evaluate whether Rothman Index variability can predict RRT^s^ activation in surgical patients	Rothman Index	RRT activation, mortality	Rothman Index variability predicted likelihood of RRT activation.
Bedoya et al, 2019 [26], United States	85,322 patients, 2 hospitals, 2014-2016	To determine the effectiveness of NEWS implementation on predicting and preventing patient deterioration	NEWS	ICU transfer or death	No change after implementing NEWS. At both academic and community hospitals, NEWS had poor performance characteristics and was generally ignored by nursing staff.
Heller et al [39], 2020, Germany	3827 patients, 2 wards, 2016-2017	To develop a prediction model for Code Blue, using EMR^t^ data, and compare with MEWS	MEWS with paging functionality	CA or ICU transfer	The rate of CA and ICU transfers significantly decreased after implementing MEWS with paging functionality.

^a^MET: medical emergency team.

^b^CA: cardiac arrest.

^c^ICU: intensive care unit.

^d^EWS: early warning score.

^e^MEWS: Modified Early Warning Score.

^f^SEWS: Standardized Early Warning Score.

^g^GMEWS: Global Modified Early Warning Score.

^h^ViEWS: VitalPAC Early Warning Score.

^i^NEWS: National Early Warning Score.

^j^RRS: rapid response system.

^k^SOFA: Sequential Organ Failure Assessment.

^l^PIRO: Predisposition, Infection, Response, Organ, Dysfunction Score.

^m^SCS: simple clinical score.

^n^MEDS: Mortality in Emergency Department Sepsis.

^o^SAPS II: Simple Acute Physiology Score II.

^p^REMS: Rapid Emergency Medicine Score.

^q^APACHE II: Acute Physiology and Chronic Health Evaluation Score II.

^r^AUC: area under the receiver operating characteristic curve.

^s^RRT: rapid response team.

^t^EMR: electronic medical record.

**Table 4:** Studies of scoring tool implementation.

Author, year, and country	Study design	Setting	Study aim	Scoring tool	Intervention assessment	Implementation outcome	Key findings or conclusions
Bailey et al, 2013 [61], United States	PS^a^: RCT^b^	19,116 patients, 1 hospital, 2007-2011	To validate the EWS^c^ in general medical wards and trial real time alerting.	Two-tiered EWS [38]	Comparison of alerts between intervention and control patients.	Among patients identified by EWS, there were no differences in proportion transferred to ICU^d^ or died in the intervention group compared with control.	Alerts generated for patients meeting the threshold were highly specific for ICU transfer and death. However, sending real time alerts to the nurse manager did not improve event outcomes.
Evans et al, 2015 [64], United States	PS: observational study	6289 patients, 1 hospital, 2012-2013	To develop and evaluate a detection and alert system for monitoring patients every 5 min	MEWS^e^ with pager alerts	Comparison of events from patients in 2 wards pre and post intervention	Ward A patients had more ICU transfers, MET^f^ calls and greater LOS^g^ but fewer deaths during intervention compared with preintervention. No differences were seen in ward B.	Implementation of the predictive model increased appropriate MET calls. Mortality decreased in the ward with older patients and multiple comorbidities, but not in the other ward.
Subbe et al, 2017, [65], United Kingdom	PS: before and after study	4402 patients, 1 hospital, 2014-2015	To assess the effect of a vital sign monitoring and alert system on outcomes	Vital sign monitoring system	Comparison of serious events between control and intervention periods	Deaths, CAs^h^ and, for patients transferred to ICU, severity of illness scores were lower in the intervention compared with control.	Deploying an EWS based on vital signs increased RRT^i^ calls and decreased mortality and CAs.
Oh et al, 2018 [52], Korea	RS^j^: before and after study	207,054 surgeries, 1 hospital, 2008-2016	To evaluate whether a RRS^k^ reduces incidence of postoperative CA	RRS with thresholds and calling criteria	Change in cardiopulmonary arrest rate in patients before and after intervention	Cardiopulmonary arrest relative risk (pre vs post intervention) was 0.56 during RRS operational hours but was unchanged during nonoperational hours. These associations remained after comorbidity adjustment.	Implementation of the RRS reduced postoperative cardiopulmonary arrest incidence but only during RRS operational hours.
Morgan et al, 2020 [50], United States	PS: quality improvement study	30,292 patients, 1 hospital, 2017-2018	To evaluate the implementation of a continuous cloud-based EWS to activate an RRT.	Cloud-based modified NEWS^l^ with RRT call threshold	Comparison of intervention with control patients for time to first lactate order, ICU^d^ transfer and mortality.	The intervention group had improved the time to the first lactate order within 24 hours of modified NEWS ≥7. There was no significant improvement in time to ICU transfer, ICU length of stay, or hospital mortality.	The study provides preliminary evidence for a pragmatic integration of cloud-based, automated monitoring with standardized and timely RRT intervention.

^a^PS: prospective study.

^b^RCT: randomized controlled trial.

^c^EWS: early warning score.

^d^ICU: intensive care unit.

^e^MEWS: Modified Early Warning Score.

^f^MET: medical emergency team.

^g^LOS: length of stay.

^h^CA: cardiac arrest.

^i^RRT: rapid response team.

^j^RS: retrospective study.

^k^RRS, rapid response system.

^l^NEWS: National Early Warning Score.

## Discussion

### Principal Findings

This review examines the use of data sets collected in EMRs to develop and implement decision support for clinicians to predict and prevent inpatient deterioration. The current literature confirms that it is possible for routinely collected EMR data to be used to anticipate patient deterioration. However, there are few reports on the performance and efficacy of these systems when used in clinical settings. Despite the wide and increasing adoption of EMRs, the successful implementation of EMR-based early warning systems or their impact on patient-centered outcomes is not commonly reported.

The studies that met the eligibility criteria and the variability in methods created a narrative review rather than a quantitative review. There was considerable variation in the models developed, methodological approach, and data collected. The common study designs (retrospective, small cohorts, and before and after studies) pose a risk of bias. The institutions where models were developed and implemented predominately from the United States, which may pose additional risks of bias because of the nature of the health care system and environment. The heterogeneity in the methodology of the developed models makes comparison of their statistical accuracy and performance difficult. The area under the receiver operating characteristic curve statistic has been used as a comparison metric, but it is important to note the differences in model specification, complexity, and outcome when interpreting model output (eg, scores) and their usefulness for implementation in a real-time clinical setting. In addition, the reporting of superior performance in statistics from models of greater complexity may be the result of model overfitting if this has not been appropriately addressed in model development. Consideration should be made such that data used to develop early warning tools reflect previous cohorts of patients and may not be relevant for future cohorts. It is reasonable that retrospective patterns of deterioration will repeat themselves in the future; however, with advances in health care technology and the potential for new and emerging health disease trends, adapting or updating tools will be required to maintain relevance. We conclude that the effectiveness of current EMR-based digital early warning tools remains promising but has not been reproducibly demonstrated.

We propose that there are multiple factors that make a patient-centered outcome evaluation of EMR-based deterioration prediction difficult. Demonstration of improved patient outcomes following early warning tool implementation relies on the successful performance of all four components of implementation, recognition, escalation, and response. Where there has been successful statistical validation for the EWSs, validation of the escalation and response (ie, implementation) has been elusive. Several factors may contribute, which are not unique to digital warning implementation. The current metrics for outcome measures are problematic. Death, an outcome that is easy to count, does not always account for treatment limitations. Cardiac arrest events are rare; therefore, they are insensitive measures. ICU transfer can reflect MET resourcing deficiencies, ICU bed availability, and local admission practices or end-of-life planning rather than preventable deterioration. Emergency callout rates may reflect individual clinician sentiment rather than true risk or deterioration, or alternatively, not be called when they should be. There is inconsistency in efferent limb performance (assessment, intervention, transfer, monitoring, and follow-up). The time required for implementation and translation into improved outcomes will frequently be confounded by other system improvements, changes within an individual health service, or the maturity of the rapid response system itself. Therefore, the relationship between implementation and patient outcomes remains unstudied, rather than unproven by the current studies.

These issues will continue to challenge the empirical validation of a complete rapid response system. The lifecycle of these technologies has yet to mature to the point where evaluation and assessment of a possible effective intervention can be performed. One example of practical limitations is to recognize which data are contemporaneously available. Data can only inform a ward-based, real-time prediction model once the information is available in the EMR for analysis. This is relevant to model development and evaluation. For a pathology result, for example, there are times of sample collection, arrival at the laboratory or analysis, and the time the result is available for integration into the clinical picture. The results of the test are only available for integration once they are available in the clinical space. However, the metadata confirming that the sample was actually ordered or collected, where it was done, and the number of samples, is immediately available. The result of a positive blood culture can take hours to days, and a definitive negative result can only be finalized 2-5 days after collection. Providing actionable decision support with such dynamic data sets is a challenge, and it could be argued that the performance of the model on an experimental data set is irrelevant unless, once deployed to the EMR, the relevant decision support is actionable by clinicians in time to avoid an adverse outcome.

As maturity grows in the development cycle within EMRs, there will be potential opportunities to improve EMR-based deterioration tools and assess their impact on patient care. The development of algorithms can help monitor in real time rich clinical data from our patients, including vital signs, investigation results, drug prescription, and provide useful decision support to improve care trajectories. These clinical data can then be used at the patient level to provide visibility of deteriorating or at-risk patients to individual clinicians, wards, clinical teams, and the MET responders, and place decision support back into the EMR to support clinicians in accurately predicting and preventing inpatient deterioration. At the system level, the data could provide feedback to guide the optimization of the digital or clinician interaction and maximize response efficiency. Successful implementation should improve patient-centered outcomes, reduce suffering, incapacity, and mortality, as well as reduce length of stay, increase hospital capacity, improve efficiency, and increase care delivery to meet ever-increasing demands.

One final, but highly important, factor for consideration in the future implementation of EMR-based deterioration tools is the aspect of medical device regulation. For example, with the recent introduction of regulations for software based medical devices in Australia by the Therapeutic Goods Administration, clinical decision support software such as algorithms to predict patient deterioration that meet the definition of a medical device would be subject to regulation and possibly inclusion in the Australian Register of Therapeutic Goods. The legislation also allows for clinical decision support software to be considered as an exempt medical device subject to how it is intended to be used. Either way, it is expected that regulation will lead to increased clinical acceptance and uptake of such algorithms.

### Conclusions

The development and accuracy of digital EWSs is increasing, facilitated by the growing availability of digital data sets. However, despite the relative performance of algorithms that can predict patient deterioration, the current literature shows that limited deployment of such algorithms into clinical practice is associated with improvement in patient outcomes. There is a paucity of quality studies in this area, and further work is needed to explore potential clinical benefits, including optimizing of the digital or clinician interaction, consideration of limitations in implementation, such as the requirement for real-time data availability, and use of standardized measures.

## Appendix

Multimedia Appendix 1 Studies of more complex patient deterioration models.

Multimedia Appendix 2 Full details of the papers included in this review, including information on prediction model input variables, performance metrics, and quality assessment.

## Abbreviations

EMR: electronic medical record
EWS: early warning score
ICU: intensive care unit
MeSH: Medical Subject Headings
MET: medical emergency team
PRISMA: Preferred Reporting in Systematic Reviews and Meta-Analyses
PROBAST: Prediction Model Risk of Bias Assessment Tool
ROBINS-I: Risk of Bias in Non-Randomised Intervention Studies
