# Supplementary material for: Predicting Patient Deterioration: A Review of Tools in the Digital Hospital Setting
Source: J Med Internet Res. 2021 Sep 30;23(9):e28209. doi: 10.2196/28209 (PMC8517822; doi:10.2196/28209)
Supplement: Multimedia Appendix 1 [file jmir_v23i9e28209_app1.docx]

Multimedia Appendix 1.

Table S1. Studies of more complex patient deterioration models.

| Author, Year, Country | Study design | Setting | Study aim | Model type | Prediction event | Key findings |
| --- | --- | --- | --- | --- | --- | --- |
| Hackmann et al, 2011 [38], United States of America | RSa: Tool development | 28,927 hospitalizations, 19,116 patients,1 hospital, 2007-2010 | To demonstrate feasibility of an EWS^h^ and validate in general medical wards with trial real time alerting. | Logistic regression, Two-tiered EWS^h^ | ICU^c^ transfer, death and length of stay | Real time alerts generated by the predictive algorithm were highly specific for ICU^c^ transfer and death but sending alerts to nurse managers did not improve intervention outcomes. Model AUC^d^ 0.88, real time simulation model 0.73. |
| Kirkland et al, 2013 [45], United States of America | RS^a^: Tool development and validation | 2213 patients, 1 hospital, 2008-2009 | To create and validate a clinical deterioration prediction tool using clinical and nursing measures. | Logistic regression with GEE^e^ approach | ICU^c^ transfer, RRT^f^ call or death | The Braden Scale, respiratory rate, oxygen saturation, and shock index were predictive of clinical deterioration 2-12 hours in the future. Model AUC^d^ 0.69, validation model 0.71. |
| Loekito et al, 2013 [47], Australia | RS^a^: Tool development and validation | 55,838 patients, 2 hospitals, 2011 | To estimate the ability of 30 laboratory variables to predict imminent death in ward patients. | Logistic regression | Death | Using combinations of commonly performed laboratory tests had fair to good predictive values for death in ward patients. Model AUC^d^ 0.87, validation model 0.88. |
| Wong et al, 2013 [58], Canada | RS^a^: Tool development | 159,794 hospitalizations, 1 hospital, 2004-2009 | To determine if post-admission time-dependent covariates improve a survival model predicting daily risk of death. | Time-dependent and time-fixed Cox regression | Time to death | All comparative measures clearly indicated that the addition of time-dependent covariates improved the prediction of daily hospital death risk. Validation model c-statistic 0.88. |
| Capan et al, 2015 [27], United States of America | RS^a^: Tool development | 38,356 patients, 1 hospital, 2011 | To identify optimal RRT^f^ activation rules using EMR^g^-derived Markovian models. | semi-Markov decision process | NEWS^j^, RRT^f^ trigger, CA^h^, ICU^c^ transfer or death | Models identified two sets of subpopulation specific RRT^f^ activation rules. The thresholds differed as a function of admission type and frailty (Braden skin score). |
| Alaa et al, 2018 [23], United States of America | RS^a^: Tool development | 6094 patients, 1 hospital,2013-2016 | To develop a new model for predicting patient deterioration: The HASMM^j^. | Hidden Absorbing Semi-Markov Model (HASMM^j^) | ICUc transfer | HASMM^j^ performed better than other clinical risk scores, predicating deterioration 8-9 hours before ICU admission. Model AUCd for precision vs recall 0.49, a 22% gain on RI^r^. |
| Alaa et al, 2018 [24], United States of America | RS^a^: Tool development | 6321 patients, 1 hospital, 2013-2016 | To develop a personalized real time risk scoring algorithm for predicting ICU^c^ admission. | Multitask Gaussian processes | ICU^c^ transfer | The algorithm significantly outperformed similar risk scores in terms of timeliness, true positive rate, specificity and positive predictive value (AUC^d^ 0.36, 0.81 and 0.61, respectively). It offered gains of 12% compared RI. |
| Redfern et al 2018 [53] UK | RS^a^: Tool development | 979,333 hospitalizations, 4 hospitals, 2014-2016 | To develop and validate an EWS^b^ that combined NEWS^j^ and LDT-EWS^k^. | NEWS^j^ and decision tree | ICU^c^ transfer or death | The LDT-EWS^k^, NEWS^j^ risk index increases the ability to identify patients at risk of deterioration compared with the NEWS^j^ alone. Validation model's AUCs^d^ 0.90 and 0.92. |
| Kwon et al 2018 [46], Cho et al 2020 [28] Korea | RS^a^: Tool development and evaluation | 60,170 patients, 2 hospitals, 2010-2019 | To develop a deep-learning early warning system and compare performance to conventional methods. | Neural net | ICU^c^ transfer, CA^h^ | DEWS^l^ identified >50% of patients with CA^h^ 14 hours before the event, and outperformed MEWS^n^, random forest, logistic and single parameter track and trigger system. Validation model's AUCs^d^ 0.85, 0.84, and evaluation model 0.86. |
| Arnold et al, 2019 [68], United States of America | PS^m^: Tool validation | 1874 patient days,  1 hospital,  2015 | To compare an automated EWS^b^ and physicians in predicating patient clinical deterioration. | Rothman index and logistic regression | ICU^c^ transfer, CA^h^ or RRT^h^ activation | No significant difference between EWS^b^ and physicians, a combined model outperformed either alone. Combined model AUC^d^ 0.78. |
| Mohamadlou et al, 2019 [49], United States of America | RS^a^: Tool development | 644,230 patients, 3 hospitals, 2008-2017 | To evaluate mortality predictions based on boosted trees. | Gradient boosted trees | Death | The model predicated mortality 48 hours in advance using only vital signs (static and time differing) AUCs^d^ 0.96, 0.95 and 0.94 for 12-, 24- and 48-hour predictions. Boosted tree model was more accurate than logistic, SVM^o^, MEWS^n,^ and qSOFA^p^. |
| Shamout et al, 2019 [55], United Kingdom | RS^a^: Tool development | 37,284 hospitalizations, 4 hospitals, 2014-2018 | To develop the deep early warning system to predict adverse events. | Gaussian process regression with attention-based neural net | CA^h^, ICU^c^ Transfer or death | DEWS^l^ had greater accuracy than NEWS^j^ and logistic regression, AUC^d^ 0.88, and had improved clinical utility and interpretability to supplement existing EWS^b^. |
| Ye et al, 2019 [60], United States of America | RS^a^/PS^m^: Tool development and evaluation | 54,246 patients, 2 hospitals, 2015-2017 | To validate a real time EWS^b^ designed to predict mortality risk. | Random forest | Death | The EWS^b^ algorithm accurately predicted death 40.8 hours earlier for the top 13% of patients at risk, model c-statistic 0.88. The EWS^b^ can assist in clinical decision making with more actionable and effective individualized care. |
| Fejza et al, 2019 [36], United States of America | RS^a^: Tool development | 1,271,733 hospitalizations, 417 hospitals, 12 months | To develop a model to predict mortality and update the prediction day by day. | Logistic regression and ensemble techniques | ICU^c^ transfer, death, HAI^q^ or pressure ulcer | Models with admitting diagnosis and drugs during hospital stay had AUCs^d^ 0.64-0.85 for day 1 outcomes. A trade-off between model complexity and accuracy using data later in hospital stay was seen with historical data becoming less predictive over time. |
| Keim-Maplass et al, 2019 [42], United States of America | RS^a^: Tool development | 8111 patients, 1 hospital,2013-2015 | To develop a model using continuous physiologic monitoring data to predict clinical deterioration. | Logistic Regression | ICU^c^ transfer or death | Event rates were 3.4-fold higher for patients with risk spikes with associated alerts firing about once a day. Risk spikes were driven by respiratory changes and alert thresholds could be used as the change from patient baseline rather than an arbitrary threshold. |
| Kia et al, 2020 [43], United States of America | RS^a^: Tool development and evaluation | 157,984 hospitalizations, 1 hospital, 2011-2017 | To describe MEWS^n^++ that enables identification of patients at risk of escalation of care or death 6 hours before the event. | Random forest | ICU^c^ transfer or death within 6 hours | The random forest model had best performance compared with logistic, SVM^o^ models and MEWS, AUC^d^ 0.88. The model can warn of patient deterioration hours before the event to help make timely decisions. |
| O'Brien et al, 2020 [51], United States of America | RS^a^: Tool development and implementation | 87,897 hospitalizations,  1 hospital,  2014-2016 | To develop, implement and evaluate a risk model for patient deterioration. | Logistic regression | ICU^c^ Transfer or death | The model had better performance than NEWS, average time-varying AUC^d^ 0.81, and was able to respond to acute clinical changes in patients’ clinical status. During implementation score thresholds needed to be retuned to obtain desired sensitivity. |

^a^RS: retrospective study.

^b^EWS: Early Warning Score.

^c^ICU: intensive care unit.

^d^AUC: area under the receiver operating characteristic.

^e^GEE: generalized estimating equation.

^f^RRT: rapid response team.

^g^EMR: electronic medical record.

^h^CA: cardiac arrest.

^i^HASMM: Hidden Absorbing Semi-Markov Model.

^j^NEWS: National Early Warning Score.

^k^LDT-EWS: Laboratory Decision Tree Early Warning Score.

^l^DEWS: deep-learning Early Warning Score.

^m^PS: prospective study.

^n^MEWS: modified Early Warning Score.

^o^SVM: support vector machine.

^p^qSOFA: quick sequential organ failure assessment.

^q^HAI: hospital acquired infection.

^r^RI: Rothman index.
