# Supplementary material for: Predicting Patient Deterioration: A Review of Tools in the Digital Hospital Setting
Source: J Med Internet Res. 2021 Sep 30;23(9):e28209. doi: 10.2196/28209 (PMC8517822; doi:10.2196/28209)
Supplement: Multimedia Appendix 2 [file jmir_v23i9e28209_app2.docx]

**Multimedia Appendix 2 - Supplementary Tables**

[Table S2 Further details of prediction models developed in studies reviewed. 2](#_Toc77185429)

[Table S3 Input variable details for patient deterioration scoring tools 8](#_Toc77185430)

[Table S4 Quality assessment of implementation studies 9](#_Toc77185431)

Table S2 Further details of prediction models developed in studies reviewed.

| **Study** | **Model Type** | **Model outcome measure** | **Input variables used in predication model (final model only)** | **Modelling data** | **Model performance metrics** | **PROBAST Quality Assessment. Risk of Bias (RoB) and Applicability Concerns (AC)** |
| --- | --- | --- | --- | --- | --- | --- |
| Escobar *et al*. 2012  [35] | Pooled logistic regression models | Transfer to ICU or death | Age, sex, care directive status, illness severity (LAPS at admission), comorbidity burden (COPS, COPS status), Length of stay, time of day, Vital signs (temperature, heart rate, respiratory rate, systolic and DBP, pulse oximetry, neurological status), Laboratory tests (blood urea nitrogen, proxy for measured lactate, haematocrit, white blood cell count) | Total: 102,442 patients' hospitalisations (4036 event shifts, 39,782 comparison shifts) Derivation model: 1979 event shifts 9509 comparison shifts Validation model: 2057 event shifts, 20273 comparison shifts | All diagnosis model: c-statistic 0.845 (0.826-0.863) in derivation dataset, 0.775 (0.753 - 0.797) in validation dataset | RoB: Low AC: Low |
| Escobar *et al*. 2013  [34] | Pooled logistic regression models | Inpatient death | Age, sex, admission venue, admission diagnosis, severity of illness (LAPS2 which includes laboratory results and vital signs), comorbidity burden (COPS2 or Charlson comorbidity Index), care directive status | Total: 391,584 hospitalisation/248,383 patients derivation model: 279,929 hospitalisations/ 187,083 patients Validation model: 111,655 hospitalisations/86,433 patients | Comprehensive model: c-statistic 0.798, Nagelkerke's pseudo-R2 0.295 in validation dataset. | RoB: Low AC: Low |
| Kipnis *et al*. 2016  [44] | Discrete time logistic regression | Transfer to ICU or death | Age, sex, illness severity (LAPS2), comorbidity burden (COPS2), LOS, prior ICU visit, care directive, season, time of day, admission category, Vital signs (systolic and DBPs, heart rate, oxygen saturation, respiratory rate, temperature, neurological status, proxy for measured lactate, shock index), Laboratory tests (Anion gap, bicarbonate, glucose, haematocrit, lactate, blood urea nitrogen, creatinine, sodium, troponin, total white blood cell count) | Total: 649,418 episodes/ 374,838 patients/ 48,723,248 hourly observations Training set 1: 24,492,639 hourly observations Training set 2: 12,042,741 hourly observations Validation set: 12,186,823 hourly observations | Final model c-statistic 0.82 (0.81 - 0.83) in validation dataset. Overall sensitivity 49%, specificity 92.2%, PPV 16.2%, NPV 98.3%.  Comparison to NEWS and CART, AAM had better sensitivity and specificity. | RoB: Low AC: Low |
| Churpek *et al*. 2012  [31] | Logistic regression | CA or Transfer to ICU | Age, Vital signs (respiratory rate, heart rate, DBP, pulse pressure index) | Total: 47427 patients all used in model derivation Validation uses the same data applying the MEWS score | Final model: AUC-ROC 0.84, at specificity of 89.9% sensitivity was 53.4. Comparison to MEWS, CART had better AUC-ROC and sensitivity. | RoB: Low AC: High |
| Churpek *et al.* 2013  [29] | Logistic regression  (4 models) | CA; Transfer to ICU; Death; All combined | Vital Signs (heart rate, systolic and DBP, respiratory rate, oxygen saturation, temperature, supplemental oxygen requirement, level of consciousness) | Total: 59643 patients  Dataset split into two halves for model derivation and validation | Validation models: Mortality outcome models AUC-ROC range 0.73-0.82 Cardiac arrest outcome models AUC-ROC range 0.74-0.76 ICU transfer outcome models AUC-ROC range 0.68-0.71 Combined outcome models AUC-ROC range 0.68-0.71 | RoB: Unclear AC: High |
| Churpek *et al.* 2014  [30] | Logistic regression | CA and Transfer to ICU | Age, prior ICU admission, time since first ward vital sign, Vital Signs (respiratory rate, DBP, heart rate, oxygen saturation, supplemental oxygen use, temperature, AVPU), Laboratory results (hemoglobulin, platelet count, white blood cell count, blood urea nitrogen, potassium, anion gap) | Total: 59 301 patients Dataset split into thirds for 3-fold cross-validation | Validation model: Cardiac Arrest outcome AUC-ROC 0.87 (0.86 - 0.88) ICU Transfer outcome AUC-ROC 0.76 (0.75-0.76) Derived model at specificity of 93% had sensitivity of 65%. Comparison to ViEWS, eCART had better sensitivity and specificity | RoB: Low AC: Low |
| Churpek *et al.* 2014  [33] | Survival analysis | CA, Transfer to ICU or death | Age, number of previous ICU stays, Vital signs (respiratory rate, heart rate, SBP, DBP, temperature, Pulse pressure index, oxygen saturation, AVPU), Laboratory values (sodium, potassium, bicarbonate, anion gap, blood urea nitrogen, creatinine, BUN to C ratio, glucose, calcium, WBC, haemoglobin, Platelets, total protein, albumin, total bilirubin, AST, alkaline phosphate) | Derivation model: 162088 patient admissions Validation model: 107911 patient admissions | Validation models: Cardiac arrest outcome AUC-ROC 0.83 ICU transfer outcome AUC-ROC 0.75 Death outcome AUC-ROC 0.93 Combined outcomes AUC-ROC 0.77 Comparison to MEWS, eCART had better AUC-ROC and specificity (at a similar sensitivity level) | RoB: Low AC: Low |
| Somanchi *et al.* 2015  [56] | SVM and Logistic regression | Code blue event in next x hours | Age, gender, race, ethnicity, past hospitalisation history (number of previous code blues, hospital discharges, emergency admissions, hospital encounters), real-time vital signs and laboratory test: 29 variables measured at different times during hospitalisation including, respiratory rate, blood pressures, pulse oximetry, temperature, haemoglobin, platelet count, haematocrit, glucose count, creatinine, potassium and sodium. | Total: 232000 hospital encounters Derivation models: start with hospital encounters up to 2011 and sequentially add a month to create 8 training datasets Validation models: Month following derivation data | Validation models: SVM models, Code blue in next 1, 2, 3 and 4 hours AUC-ROC range 0.76 - 0.78 Comparison to MEWS, SVM had higher recall and AUC-ROC | ROB: Low AC: Unclear |
| Churpek *et al.* 2016  [32] | Logistic, decision trees, SVM, K-NN, Neural net, MEWS | CA, Transfer to ICU or death | Age, time since ward admission, number of previous ICU stays, Vital signs (respiratory rate, heart rate, SBP, DBP, temperature, Pulse pressure index, oxygen saturation, AVPU), Laboratory values (electrolytes, creatinine, liver function tests, and blood counts). | Total: 269 999 patient admissions Derivation model: First 60% of data Validation model: Following 40% of data | Validation models AUC-ROC: Random Forest 0.801, Gradient boosted machine 0.794, Bagged trees 0.789, SVM 0.786, Neural network 0.782, Logistic (spline) 0.770, K-NN 0.754, Logistic (linear) 0.735, Decision tree 0.734 and MEWS 0.698 | RoB: Low AC: Low |
| Mayampurath *et al.* 2019  [48] | Convolutional neural network | Death | Age, sex, race, prior cardiac arrest, prior admission within 90 days, hour of day, Vital signs (temperature, heart rate, respiratory rate, SBP, DBP, O2 Saturation, FiO2, AVPU), Laboratory results (Sodium, Potassium, Bicarbonate, Anion Gap, Glucose, Calcium, Blood Urea Nitrogen (BUN), Serum Creatinine (SCr), BUN/SCr ratio, Phosphate, liver function test, complete blood count), Medications (grouped into medication type), interventions (e.g. mechanical ventilation, dialysis), morse and Braden scores and diagnostic tests (e.g. chest xrays, EKG, CT scans). | Total: 115825 hospital admissions Derivation model: 70% of data Validation model: 30% of data | Validation models AUC-ROC: Standard CNN 0.87, Recurrent NN 0.89, Deep-CNN 0.90, CNN-Recurrent Layer 0.90 Comparison to SOFA (AUC-ROC 0.57) and MEWS (AUC-ROC 0.76), all neural networks had better model accuracy | RoB: Low AC: Low |
| Hackmann *et al.* 2011  [38] | Logistic regression, Two-tiered EWS | ICU transfer, death and LOS | Age, Charlson Index, Vital Signs (DBP, SBP, Oxygen Saturation pulse oximetry, Pulse, Respirations, Shock Index, Temperature), Laboratory values (Alanine aminotransferase, Anion gap, Aspartate aminotransferase, serum Calcium, serum ionized Calcium, Estimated creatinine clearance, serum Magnesium, serum Phosphate, serum Potassium). | Total: 28927 hospital encounters Derivation model: One single 24-hour window of data per patient Validation model: 2nd half of patient data | Validation model: AUC-ROC 0.7293, specificity 0.9492, sensitivity 0.4127, PPV 0.2955, NPV 0.9691, accuracy 0.9229 | RoB: Unclear AC: Low |
| Kirkland *et al.* 2013  [45] | Logistic regression with GEE approach | ICU transfer, RRT call or death | Respiratory rate, Oxygen saturation, shock index and Braden scale | Derivation model: 276 patients Validation model: 1946 patients | Validation model: AUC-ROC 0.71 (0.68 - 0.74) | RoB: High AC: Unclear |
| Loekito *et al.* 2013  [47] | Logistic regression | Death | Age, urea, creatinine, total bicarbonate concentration, bilirubin, albumin concentration, haematocrit, white cell count | Derivation model: 40062 batches of laboratory measurements Validation model: 118999 batches of laboratory measurements | Validation model: AUC-ROC 0.87 (0.85 - 0.89) | RoB: Low AC: Unclear |
| Wong *et al.* 2013  [58] | Time-dependent and time-fixed Cox regression | Time to death | Age, admission type, Exhauster score, Laboratory-based Acute Physiology Score (LAPS), ICU status, alternate level of care, Procedural Index for Mortality Risk (PIMR) score | Derivation model: 106522 hospitalisations Validation model: 53265 hospitalisations | Validation models: Time-fixed AUC-ROC 0.811 (0.802 - 0.280) Time-dependent AUC-ROC 0.879 (0.872 - 0.886) | RoB: Low AC: Low |
| Capan *et al.* 2015  [27] | semi-Markov decision process | NEWS-based RRT trigger, CA, ICU transfer or death | Admission type, SBP, heart/pulse rate, oxygen saturation, respiratory rate, supplemental oxygen, temperature, level of consciousness (AVPU), Braden skin score | Derivation model: 38356 patients Validation model: Nil | NA | RoB: Unclear AC: Unclear |
| Alaa *et al.* 2018  [23] | HASMM | ICU transfer | Age, gender, transfer status, Vital Signs (SBP, DBP, heart rate, respiratory rate, temperature, oxygen saturation, oxygen device assistance, Glasgow Coma Scale, eye opening, best motor response, best verbal response), Laboratory tests (glucose, urea nitrogen, white blood cell count, creatinine, haemoglobin, platelet count, potassium, sodium, total bicarbonate concentration, chloride) | Derivation model: 4939 patients  Validation model: 1155 patients | Validation model TPR/PPV-ROC 0.489, Timeliness 8hrs 34min Comparison to MEWS, SOFA, APACHE, Rothman index as well as other machine learning models where TPR/PPV-ROC was better. | RoB: Low AC: Low |
| Alaa *et al.* 2018  [24] | Multitask Gaussian processes | ICU transfer | Age, gender, transfer status, transplant, floor ID, ICD-9 code, race, ethnicity, Vital Signs (SBP, DBP, heart rate, respiratory rate, temperature, oxygen saturation, oxygen device assistance, Glasgow Coma Scale, eye opening, best motor response, best verbal response), Laboratory tests (glucose, urea nitrogen, white blood cell count, creatinine, haemoglobin, platelet count, potassium, sodium, total bicarbonate concentration, chloride) | Derivation model: 5130 patients Validation model: 1191 patients | Validation model AUC-ROC 0.806 Comparison to MEWS, SOFA, APACHE II and Rothman index where AUC-ROC and TPR/PPV-ROC were better. | RoB: Low AC: Low |
| Redfern *et al.* 2018  [53] | NEWS and decision tree | ICU transfer or death within 24 hours of admission | Vital Signs (SBP, heart rate, respiratory rate, body temperature, neurological status (AVPU or Glasgow Coma Scale), oxygen saturation, supplementary oxygen, observation time), Laboratory results (albumin, creatinine, haemoglobin, potassium, sodium, urea, white cell count, time since observation). | Derivation model: 97933 admissions Validation model: 21028 and 16383 admissions | Validation models AUC-ROC 0.901 and 0.916 Comparison to NEWS, models had higher discrimination | RoB: Low AC: Low |
| Kwon *et al.* 2018  [46] and Cho *et al.* 2020  [28] | Neural net | ICU transfer, CA | Vital signs (SBP, heart rate, respiratory rate and temperature). | Derivation model: 2769324 vital sign records Validation models: 152587 and 60782 vital sign records | Validation models AUC-ROC: Cardiac arrest 0.850 and 0.837, Death 0.926 and 0.911 Comparison to MEWS, random forest and logistic regression, models had higher discrimination | RoB: Low AC: Unclear |
| Mohamdalou *et al.* 2019  [49] | Gradient boosted trees | Death | Age, Vital signs (heart rate, respiratory rate, oxygen saturation, temperature, SBP, DBP, Glasgow Coma Scale). | Total: 24614 patients, 46980 patients and 32718 patients Derivation models: four-fifths of patients randomly selected per model  Validation models: one-fifth of patients randomly selected per model | Validation models average AUC-ROC:  12-hour model 0.96, 24-hour model 0.96, 48-hour model 0.96 Higher performance compared to MEWS, SOFA and other machine learning models | RoB: Low AC: Low |
| Shamout *et al.* 2019  [55] | Gaussian process regression with attention-based neural net | CA, ICU Transfer or death | Age, gender, time since admission, comorbidity (Elixhauser comorbidity index, frailty scores), Vital Signs (SBP, heart rate, respiratory rate, temperature, AVPU or GCS, peripheral oxygen saturation, fraction of inspired oxygen), Laboratory results (albumin, bilirubin, C-Reactive protein, haemoglobin, platelets, white blood cells, potassium, sodium, urea, creatinine). | Derivation model: 21512 admissions Validation model: 15772 admissions | Validation model AUC-ROC 0.880, sensitivity 0.729 Compared to NEWS and other machine learning models, performance was best | RoB: Low AC: Low |
| Ye *et al.* 2019  [60] | Random forest | Death | Age, gender, patient estimated cost last year, emergency visits last year, inpatient admissions last year, inpatient length of days last year, outpatient visits last year, disorders of lipid metabolism, anaemia, cardiovascular diseases, renal failure, type 2 diabetes, Vital Signs (SBP, DBP, pulse rate, respiratory rate, temperature, oxygen saturation), Laboratory tests (sodium, glucose, haematocrit, potassium, red blood cell count, white blood cell count, blood urea nitrogen, chloride, creatinine, haemoglobin, anion gap, platelets, estimated glomerular filtration rate) | Derivation model: 42484 inpatient encounters Validation model: 11762 hospitalisations | Validation model AUC-ROC 0.884 (Random Forest) Compared to other machine learning models, this was the highest | RoB: Unclear AC: Unclear |
| Fejza *et al.* 2019  [36] | Logistic regression and ensemble techniques | ICU transfer, death, hospital acquired infection or pressure ulcer | Age, gender, admission type, admitting diagnoses (ICD-9-CM), procedures performed during hospital stay, drugs administered | Total: 1271733 hospitalisations Derivation model: 7088373 instances Validation model: 176868 instances | Validation models: Mortality AUC-ROC 0.804, TPR/PPV-ROC 0.142 ICU Transfer AUC-ROC 0.64, TPR/PPV-ROC 0.059 | RoB: Unclear AC: Unclear |
| Keim-Maplass *et al.* 2019  [42] | Logistic Regression | ICU transfer or death | Heart rate, respiratory rate, pulse oximetry, non-invasive blood pressure, ECG-derived respiration rate, local dynamics score, density score, sample entropy, detrended fluctuation analysis of heart inter-beat intervals (DFA), Vital signs from flow sheets (pulse rate, respiratory rate, oxygen saturation, blood pressure, temperature, Glasgow Coma Scale), Laboratory results (frequently available - no detail). | Total: 8111 admissions Derivation model: 4052 admissions Validation model: 4059 admissions | Validation model AUC-ROC not reported | RoB: Low AC: Unclear |
| Kia *et al.* 2020  [43] | Random forest | ICU transfer or death within 6 hours | Age, unit type, unit length of stay, speciality unit, admission source, admission type, height, weight, cardiac regularity, level of activity, respiratory pattern, tenderness, Vital signs (SBP, DBP, pulse rate, respiratory rate, temperature, oxygen saturation, level of consciousness), Laboratory results (white blood cells, blood urea nitrogen, ABG, creatinine, lactate, chloride, haematocrit, platelet count, sodium, haemoglobin, potassium, INR, bilirubin, estimated glomerular filtration rate) | Total: 117884 bed movements Derivation model: 15818 instances Validation model: 102066 instances | Validation model AUC-ROC 0.85, TPR/PPV 0.37, sensitivity 81.6%, specificity 75.5% Compared to MEWS, logistic regression and a SVM, the sensitivity, specificity and AUC-ROC were all higher. | RoB: Low AC: Low |
| O'Brien *et al.* 2020  [51] | Logistic regression | ICU Transfer or death | Age, race, sex, time from admission, patient location, Vital signs (respiratory rate, SBP, DBP, pulse rate, temperature, level of consciousness, oxygen saturation, supplemental oxygen), Comorbidities (Chronic kidney disease, COPD, diabetes, HIV, malignancy, myocardial infarction, stroke, transplant), medication therapeutic class, Laboratory tests (bandemia, albumin, ALT, ammonia, anion gap, AST, blood cultures, blood urea nitrogen, CK, CKMB, C-Reactive protein, D-dimer, ESR, fibrinogen, haematocrit, INR, potassium, lactate, LDH, Magnesium, partial pressure of carbon dioxide, pH, platelet count, sodium, total bilirubin, troponin, white blood cells) | Derivation model: 73215 patients Validation model: 14682 patients (last 6 months of data) | Validation model average AUC-ROC 0.814 (0.79 - 0.83) Compared to NEWS, AUC-ROC is higher | RoB: Low AC: Low |

Table S3 Input variable details for patient deterioration scoring tools

| **Scoring Tool** | **Input Variables** | **Studies used in**  (see references) |
| --- | --- | --- |
| Acute Physiology and Chronic Health Evaluation II (APACHE II) | Age, history of severe organ failure, temperature, mean arterial pressure, blood pH, heart rate, respiratory rate, sodium, potassium, creatinine, haematocrit, white blood cell count, acute renal failure, Glasgow Coma Scale, fraction of inspired oxygen | [24. 59] |
| Chronic Respiratory Early Warning Score (CREWS) | Systolic blood pressure, heart/pulse rate, oxygen saturation, respiratory rate, supplemental oxygen, temperature, level of consciousness (AVPU) | [65] |
| Global Modified Early Warning Score (GMEWS) | Systolic blood pressure, heart rate, respiratory rate, temperature, neurological status (AVPU) | [54] |
| Medical alert system | Systolic blood pressure, pulse rate, respiratory distress, metabolic acidosis, partial pressure of oxygen, glucose, mental status, o2 therapy, seizure observation, chest pain, airway observation, code blue | [41] |
| MET call criteria | Systolic blood pressure, pulse rate, respiratory rate, oxygen saturation | [67] |
| Modified Early Warning Score (MEWS) | Systolic blood pressure, heart rate, respiratory rate, temperature, neurological status (AVPU) | [24, 25, 31, 33, 37, 39, 54, 59, 64] |
| modified NEWS (mNEWS) | Systolic blood pressure, heart/pulse rate, oxygen saturation, respiratory rate, supplemental oxygen, temperature | [50] |
| Mortality in Emergency Department Sepsis (MEDS) | Age, metastatic malignancy, nursing home resident, pneumonia, respiratory rate, oxygen saturation, altered mental status, white blood bands, platelets, septic shock | [59] |
| National Early Warning Score (NEWS), | Systolic blood pressure, heart/pulse rate, oxygen saturation, respiratory rate, supplemental oxygen, temperature, level of consciousness (AVPU) | [25-27, 37, 54, 65] |
| Predisposition/Infection/Response/Organ Dysfunction Score (PIRO) | Age, metastatic malignancy, nursing home resident, pneumonia, respiratory rate, heart rate, systolic blood pressure, oxygen saturation, white blood bands, platelets, blood urea nitrogen, lactate, chronic pulmonary obstructive disease, chronic liver disease | [59] |
| Rapid Emergency Medicine Score (REMS) | Age, respiratory rate, temperature, heart rate, mean arterial pressure, oxygen saturation, Glasgow Coma Scale | [59] |
| Rapid Response System (RRS) alert | Systolic blood pressure, heart rate, respiratory rate, temperature, oxygen saturation, blood pH level, partial pressure of carbon dioxide, partial pressure of oxygen, lactic acid, total bicarbonate concentration | [52] |
| Rothman Index | Systolic blood pressure, diastolic blood pressure, temperature, oxygen saturation, respiratory rate, heart rate, creatinine, sodium, chloride, potassium, blood urea nitrogen, white blood cell count, haemoglobin, cardiac rhythm, nursing assessments (cardiac, respiratory, gastrointestinal, genitourinary, neurological, skin, safety, peripheral vascular, food/nutrition, psychosocial, musculoskeletal) , Braden score. | [23, 24, 57, 68] |
| Sequential Organ Failure Assessment (SOFA) Score | Mean arterial pressure, partial pressure of oxygen, fraction of inspired oxygen, total bilirubin, Glasgow Coma Scale, platelets, creatinine | [24, 59] |
| Simple Clinical Score (SCS) | Age, nursing home resident, respiratory rate, temperature, heart rate, systolic blood pressure, oxygen saturation, altered mental status, coma, functional status, shortness of breath, abnormal EKG, new stroke, diabetes | [59] |
| Simplified Acute Physiology Score II (SAPS II) | Age, type of admission, metastatic malignancy, AIDS, temperature, heart rate, systolic blood pressure, partial pressure of oxygen, total bilirubin, Glasgow Coma Scale, white blood cells, sodium, potassium, bicarbonate, blood urea nitrogen | [59] |
| Standardised Eary Warning Score (SEWS) | Systolic blood pressure, heart rate, respiratory rate, temperature, neurological status (AVPU) | [54] |
| VitalPAC Early Warning Score (ViEWS) | Systolic blood pressure, pulse rate, respiratory rate, temperature, oxygen saturation, inspired oxygen type, neurological status (AVPU) | [54, 59] |
| Worthing | Systolic blood pressure, pulse rate, ventilatory frequency, temperature, oxygen saturation, level of consciousness (AVPU) | [54] |

Table S4 Quality assessment of implementation studies

| **Study** | **ROBINS-1 Quality Assessment** | **Bias due to confounding** | **Bias due to selection of participants** | **Bias in classification of interventions** | **Bias due to deviations from intended interventions** | **Bias due to missing data** | **Bias in measurement outcomes** | **Bias in selection of the reported result** |
| --- | --- | --- | --- | --- | --- | --- | --- | --- |
| Bailey *et al.* 2013  [61] | Low | Low | Low | Low | Low | Moderate | Low | Low |
| Evans *et al.* 2015  [64] | Low/ Moderate | Moderate | Moderate | Low | Low | Low | Low | Low |
| Subbe *et al.* 2017  [65] | Low/ Moderate | Moderate | Low | Low | Low | Moderate | Low | Low |
| Oh *et al.* 2018  [52] | Moderate | Moderate | Moderate | Low | Low | Low | Low | Low |
| Morgan *et al.* 2020  [50] | Moderate | Low | Moderate | Low | Low | Moderate | Low | Moderate |
| Escobar *et al*. 2020  [63] | Low | Low | Low | Low | Low | Low | Low | Low |
| O'Brien et al 2020  [51] | Low | Low | Low | Low | Moderate | Low | Low | Low |
